# Phytochemical and Antibacterial Investigations of the Extracts and Fractions from the Stem Bark of *Hymenaea stigonocarpa* Mart. ex Hayne and Effect on Ultrastructure of *Staphylococcus aureus* Induced by Hydroalcoholic Extract

**DOI:** 10.1155/2013/862763

**Published:** 2013-12-14

**Authors:** Gustavo Santiago Dimech, Luiz Alberto Lira Soares, Magda Assunção Ferreira, Anne Gabrielle Vasconcelos de Oliveira, Maria da Conceição Carvalho, Eulália Azevedo Ximenes

**Affiliations:** ^1^Laboratório de Fisiologia e Bioquímica de Microorganismos, Departamento de Antibióticos, Centro de Ciências Biológicas, Universidade Federal de Pernambuco, 50670-901 Recife, PE, Brazil; ^2^Laboratório de Farmacognosia, Departamento de Farmácia Universidade Federal de Pernambuco, Centro de Ciências da Saúde, 50670-901 Recife, PE, Brazil; ^3^Laboratório de Biologia Celular e Ultraestrutura, Centro de Tecnologias Estratégicas do Nordeste (CETENE), 50670-901 Recife, PE, Brazil

## Abstract

The aim of this study was to investigate the antimicrobial activity of different extracts and fractions obtained from *Hymenaea stigonocarpa* stem barks. The cyclohexanic, ethyl acetate, ethanol, aqueous, and hydroalcoholic extracts were obtained by maceration. The hydroalcoholic extract was partitioned, which resulted in the ethyl acetate and aqueous fractions. All extracts and fractions were subjected to phytochemical screening and evaluation of total phenol and tannin contents. An HPLC-DAD and ultrastructural alterations analysis were performed. Terpenes and coumarins were detected in the cyclohexanic extract. Flavonoids and condensed tannins were present in the other extracts and fractions. The extracts with the highest contents of tannins, ethanol (EE), hydroalcoholic (HE), and aqueous fraction (AF) showed also the highest antimicrobial activity. The MIC values ranged from 64 to 526 *µ*g/mL. The chromatographic fingerprints suggest the presence of astilbin and other flavonoids in EE and HE. Presence of the thick cell wall, undulating outer layer, abnormal septa, and leakage of the cytoplasmic contents and absence of cell wall and cell lyses were the main alterations observed on *Staphylococcus aureus* ATCC 33591 after treatment with the *Hymenaea stigonocarpa* hydroalcoholic extract. The presence of phenolic compounds like flavonoids and tannins is possibly the reason for the antimicrobial activity.

## 1. Introduction

Nosocomial infections caused by a multidrug resistant phenotype posed a major public health problem in the last decades [1]. These infections cause a prolonged period of hospitalization and high mortality rates [2].

It is estimated that nearly 16 billion dollars are being spent annually to treat the nosocomial infections caused by multidrug resistant microorganisms, whose therapy is limited to the use of few antimicrobial agents, which are often ineffective [3]. Indeed, the search of antimicrobial agents is an important strategy for the establishment of new alternative therapies in infections, which are difficult to handle [4].

Natural products derived from bacteria, algae, plants, and animals are known to produce a variety of secondary metabolites and are promising sources of new therapeutic agents including antimicrobials [5].

Approximately 60 to 80% of the world's population still relies on traditional medicines for treatment of several illnesses. In Brazil, plants are used as natural medicine mainly by low-income populations and contribute significantly to primary health care [6].

*Hymenaea stigonocarpa* Mart. ex Hayne (Fabaceae) is a medicinal plant found in the Brazilian savannah and popularly known as “Jatobá-do-cerrado” [7]. Its stem barks are widely used in infusion or decoction to treat stomach pain, asthma, bronchitis, ulcers, diarrhea, flu, and cough [8–10].

Phytochemical studies of *Hymenaea stigonocarpa* showed the presence of terpenes and sesquiterpenes, fatty acids, flavonoids and tannins. These metabolites are recognized by its biological activities [11–13].

Little research has focused on the pharmacological activity from *Hymenaea stigonocarpa* stem barks.

The antidiarrheal activity and healing properties of the *H. stigonocarpa* stem bark methanolic extract were recently demonstrated on experimental gastric and duodenal ulcers [13]. However, studies on the antimicrobial activity from *Hymenaea stigonocarpa* stem barks have not yet been published.

Based on this report, the aim of the present work was to perform a phytochemical and antibacterial study of extracts and fractions from the *Hymenaea stigonocarpa* stem bark to establish the fingerprint profile by HPLC-DAD and to identify the ultrastructural alterations caused by the hydroalcoholic extract on *Staphylococcus aureus* methicillin resistant strain.

## 2. Material and Methods

### 2.1. Plant Material

Stem barks from *Hymenaea stigonocarpa* Mart. ex. Hayne were collected in Camocim de São Félix city, Pernambuco, Brazil (latitude 8°21′30.00′′S and longitude 35°47′56.70′′W). A voucher specimen was deposited in the herbarium Dárdano de Andrade Lima-Instituto Agronômico de Pernambuco (IPA) and registered under number 53563.

### 2.2. Extracts Preparation

The stem barks were air-dried, powdered using a hammer mill (Tigre ASN5), and stored in amber bottles at room temperature. Serial exhaustive maceration 50 g (1 : 2 w/v) using cyclohexane (CE), ethyl acetate (EAE), ethanol (EE), and water (AE) was performed. A hydroalcoholic extract (HE) was also obtained by maceration of 60 g (1 : 2 w/v) in ethanol/water (1 : 1). The hydroalcoholic extract dry residue (5 g) was partitioned (1 : 1) with water and ethyl acetate to obtain the aqueous (AF) and ethyl acetate (EAF) fractions. All extracts and fractions were filtered through a Whatman No. 1 paper filter and evaporated at 50°C under reduced pressure, except the aqueous extract and fraction, which were lyophilized.

### 2.3. Phytochemical Screening

The phytochemical screening was carried out by thin layer chromatography (TLC) on silica gel (Merck, Germany), following the procedures described by Wagner and Harborne [14, 15]. 15 *μ*L aliquots of each extract solution (0.5% w/v) were applied to evaluate the presence of monoterpenes, sesquiterpenes, triterpenes, steroids, alkaloids, iridoids, coumarins, saponins, hydrolyzed and condensed tannins, phenylpropanoids, flavonoids, and sugars.

#### 2.3.1. Total Phenol Contents

Total of phenol contents (TPC) was performed in accordance with Folin-Ciocalteu method, as described in the European Pharmacopoeia [16].

The extracts and fractions of the *Hymenaea stigonocarpa* stem bark were solubilized in ethanol (10%) to obtain a final concentration of 0.5 mg/mL. Five milliliters of each solution was diluted to 25 mL with distilled water. From these solutions, 2 mL was transferred to a 25 mL volumetric flask containing distilled water (10 mL) and added to 1 mL of undiluted Folin-Ciocalteu reagent, so the volume was made to 25 mL with 10% aqueous Na_2_CO_3_ (w/v). The blend was left at room temperature for 30 min. Then, absorbance of the samples was read at 760 nm in an UV/Vis spectrophotometer (Evolution 60S, Thermo Scientific). Distilled water was used as a blank.

TPC was determined from the extrapolation of the calibration curve (*y* = 0.1037*x* + 0.0024, *R* ^2^ = 0.9992), which was obtained from gallic acid (Sigma Chemical Co., St. Louis, USA) solution (2.0–8.0 *μ*g/mL).

The determination of total phenol contents was carried out in triplicate. The TPC was expressed as milligrams of gallic acid equivalents (GAE) per gram of dried samples.

#### 2.3.2. Total Tannin Contents

In order to determine the total tannins, casein (100 mg) was added to 10 mL of each extract or fraction solution and homogenized for 60 min. These blends were filtered through a Whatman No. 1 paper filter. The procedure for the determination of tannins was similar to that described above.

The phenol contents in the nontannin fractions were calculated by standard curve of gallic acid. The total tannin content was calculated as follows: total tannins = total phenols − nontannin phenols [17].

### 2.4. High Performance Liquid Chromatography Analysis

Chromatographic fingerprints of *Hymenaea stigonocarpa* stem bark extracts and fractions that present the highest tannin contents and antibacterial activity (EE, HE, and AF) were obtained by high performance liquid chromatography coupled with diode array detection (HPLC-DAD).

Extracts and fractions were solubilized in methanol to obtain solutions at the 400 *μ*g/mL concentration. Astilbin solution was used as a standard at a concentration of 25 *μ*g/mL. Then, these solutions were filtered through a 0.20 *μ*m polytetrafluoroethylene membrane (Chromafil) and stored in amber vials.

Samples analysis was performed on a HPLC-DAD device (Ultimate 3000, Thermo Scientific), equipped with binary pump HPG-3x00RS, 3000 (RS) diode array detector and ACC-3000 autosampler.

Separations were achieved with a column (C18), 250 × 4.6 mm i.d., 5 *μ*m (Acclaim 120, Dionex, equipped with a guard column (C18, 4 × 3 mm i.d. Phenomenex).

The mobile phase was composed of ultrapure water (eluent A, MilliQ) and acetonitrile (eluent B, Tedia), both acidified with 0.1% trifluoroacetic acid solution and ranging from 10–23% in 35 min to 23–90% in 50 min of eluent B at a low rate of 1 mL/min. The Chromeleon 6.0 (Dionex) software was used for data acquisition and processing.

### 2.5. Determination of Antibacterial Activity

#### 2.5.1. Bacterial Strains and Inocula Standardization

Bacteria (*n* = 28) were obtained from stock cultures and maintained at our laboratory (Laboratório de Fisiologia e Bioquímica de Microorganismos (LFBM)).

For this study we used the following bacterial strains isolated from clinical specimens:* Staphylococcus aureus* (LFBM 05, LFBM 11, LFBM 13, LFBM 15, LFBM 16, LFBM 25, LFBM 26, LFBM 28, LFBM 29, and LFBM 30); *Enterococcus faecalis *(LFBM 02, LFBM 07, LFBM 08, and LFBM 18); *Klebsiella pneumoniae *LFBM 01; *Pseudomonas aeruginosa* LFBM 01; *Proteus mirabilis *LFBM 01; and LFBM 02;* Salmonella enterica* LFBM 01;* Shigella sonnei *LFBM 01 and* Vibrio cholerae* LFBM 01.

*Staphylococcus aureus* ATCC 25923, *Staphylococcus aureus* ATCC 33591, *Klebsiella pneumoniae* ATCC 700603, *Pseudomonas aeruginosa* ATCC 9027, *Escherichia coli* ATCC 25922, and *Escherichia coli* ATCC 35218 were used as standard strains.

These microorganisms were cultured onto Mueller-Hinton agar (MHA) (Acumedia Manufacturers, Baltimore, USA) and incubated at 37°C for 18 hours. Single colonies were selected and inoculated into Mueller-Hinton broth (Acumedia Manufacturers, Baltimore, USA) with turbidity comparable to that of 0.5 McFarland standards, which is equivalent to a bacterial count of approximately 10^8^ CFU/mL. In sequence, these bacterial suspensions were diluted to obtain a final inoculum of 10^7^ CFU/mL.

#### 2.5.2. Antibacterial Agents

Eurofarma Laboratório and Sanofi-Aventis provided oxacillin, ciprofloxacin, teicoplanin, and polymyxin B, respectively. Resistance was defined for each case: oxacillin (OXA, MIC ≥ 4 *μ*g/mL), ciprofloxacin (CIP, MIC ≥ 1 *μ*g/mL), teicoplanin (TEI ≥ 32), and polymyxin B (POL, MIC ≥ 8 *μ*g/mL) according to the criteria established by the Clinical Laboratory Standard Institute [18].

The CE and EAE were solubilized in dimethyl sulfoxide/Tween80/water (1/1/8), EE, AE, HE, EAF, and AF in ethanol/water (1/9) while the antimicrobial agents were solubilized in water. The stock solutions were sterilized by filtration through a Millipore membrane with a 0.22 mM porosity.

#### 2.5.3. Antibacterial Activity

The minimal inhibitory concentration (MIC) determination was performed by broth microdilution method, following the recommendations established by CLSI 2012, with some modifications. Serial twofold dilutions of extracts, fractions, and antimicrobial agents were prepared in sterile 96-well microplates containing Mueller-Hinton broth (MHB). Five microliters of the bacterial suspension was inoculated in each well to give a final concentration of 10^4^ CFU. The concentrations ranged from 1024 to 0.125 *μ*g/mL for all antimicrobial agents. Wells containing Mueller-Hinton broth with added solvents were used as a negative control. The plates were incubated at 37°C for 24 h. Bacterial viability was detected by adding 20 *μ*L of 2,3,5-triphenyl-2H-tetrazolium chloride (TTC) 0.5% in aqueous solution. The plates were reincubated at 36°C for 2 h, and in those wells where bacterial growth occurred the TTC changed to red. The absence of a color change indicated absence of growth. MIC was defined as the lowest concentration of antibacterial agents that inhibited visible growth, as indicated by TTC staining. All experiments were carried out in duplicate.

### 2.6. Electron Transmission Microscopy

*Staphylococcus aureus* ATCC 33591 was cultured in Mueller-Hinton broth (control) and in Mueller-Hinton broth added to the *Hymenaea stigonocarpa* hydroalcoholic extract at 256 or 128 *μ*g/mL (MIC and 1/2 MIC), respectively.

The cultures were incubated at 37°C for 24 hours. These cultures were centrifuged and the pellets were fixed in 4% paraformaldehyde and 2.5% glutaraldehyde in 0.1 M sodium cacodylate buffer (pH 7.2) for 4 hours at room temperature.

Then, pellets were washed and centrifuged for 10 minutes in the same buffer. This procedure was repeated three times. The pellets were postfixed in 2% osmium tetroxide, 0.8% potassium ferricyanide, and 5 mM calcium chloride in 0.1 M sodium cacodylate buffer for 1hour and washed in sodium cacodylate buffer. The samples were dehydrated through a gradient of acetone solutions and embedded in Epon 812 resin (Sigma, USA). Subsequently, ultrathin sections were obtained using an ultramicrotome (Leica UC6), stained with uranyl acetate and lead citrate, and then examined under a Morgani-FEI transmission electron microscope (TEM).

### 2.7. Statistical Analysis

The statistical analysis was made through a one-way ANOVA and Newman-Keuls multiple comparison test, using GraphPad Prism version 5.0 for Windows, GraphPad software (San Diego, CA, USA), and *P* < 0.05 was used as the level of significance.

## 3. Results and Discussion 

### 3.1. Phytochemical Screening

The phytochemical profile of the *Hymenaea stigonocarpa* stem bark showed the presence of terpenes and coumarins in the cyclohexanic extract (CE), flavonoids and condensed tannins in ethyl acetate (EAE), and ethanolic (EE), aqueous (AE), and hydroalcoholic (HE) extracts in the ethyl acetate fraction (EAF). The aqueous fraction (AF) showed only the presence of condensed tannins. These results are in accordance with studies that demonstrated the presence of flavonoids, terpenes, steroids, and tannins in the heartwood, stem bark, and seeds of *Hymenaea stigonocarpa* [11–13, 19].

The presence of terpenes, steroids, and mainly polyphenols was described previously in *Hymenaea courbaril*, *Hymenaea parvifolia*, *Hymenaea palustris*, and *Hymenaea martiana* [20–24].

The yields expressed regarding dry weight plant material, as well as the total phenols and tannin contents, are shown in Table 1. The yield of the extracts and fractions varied from 57.0 ± 5.00 to 0.35 ± 0.02%, with a descending order of AF > EAF > HE > EE > EAE > AE > CE.

The partition of hydroalcoholic extract with water resulted in the highest amount of total extractive compounds. Total phenols content ranged from 4.95 ± 0.02 to 7.12 ± 0.03 mg GAE/g of dried samples. These results indicate a high content of these bioactive compounds in the polar extracts. The TPCs are about 20 to 29 times higher than the value found in ethanolic extract from *Hymenaea stigonocarpa* wood [19].

### 3.2. HPLC-DAD Analysis

**Figure 1:**
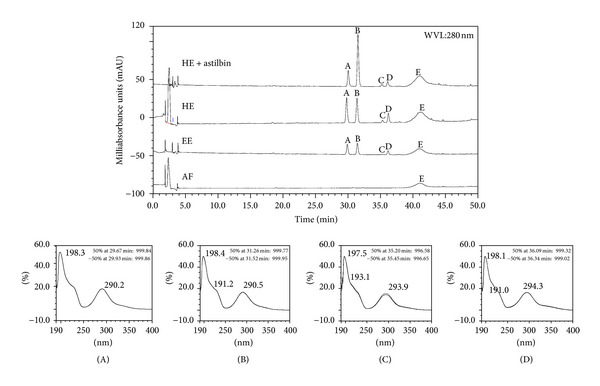
Chromatographic fingerprints from *Hymenaea stigonocarpa* stem barks. Hydroalcoholic extract (HE) + astilbin (98%), hydroalcoholic extract (HE), ethanolic extract (EE), and aqueous fraction (AF) obtained by HPLC DAD at 280 nm. Each chromatographic peak appears with its corresponding retention time. UV spectrum pattern derivatives flavonoids (A, C, and D) identified as astilbin (B).

The EE, HE, and AF that showed the highest tannin contents were selected to be analyzed by HPLC-DAD. The chromatography fingerprints of these extracts are showed in Figure 1.

Peaks (A, B, C, and D) presented in all chromatograms, eluted in the retention time from 29 to 36 min are suggestive of flavonoid derivatives.These resultats are in accordance with literature data [25].

Peak B (*R*_*t*_ = 31.453) was identified as astilbin. This identification was possible because of the comparison between the absorption spectra of the EE, HE, and HE + astilbin, whose maximum absorption was 290.3 nm.

The hump E (*R*_*t*_ 39–48) seems to be related to condensed tannins observed in this work on TLC analysis. Only monomers and oligomers up to tetramers of catechins and proanthocyanidins can be separated and detected as a defined peak [26, 27]. Polymeric catechins present in many plant materials do not result in well defined peaks. They allow a drift to happen in the baseline and the formation of characteristic humps in HPLC chromatograms.

### 3.3. Antibacterial Activity

MIC values of *Hymenaea stigonocarpa* extracts, fractions, and antimicrobial agents against *Staphylococcus aureus* and *Enterococcus faecalis *strains are listed in Table 2.

Among all microorganisms, the *Staphylococcus aureus* strains showed to be more sensitive to *H. stigonocarpa *extracts and fractions. Gram-negative bacilli showed resistance to all extracts and fractions evaluated (MIC ≥ 1024 *μ*g/mL). This resistance may be due to the presence of lipopolysaccharide, which would have blocked the interaction between phytochemicals present in the extract and the outer membrane of the microorganism [28].

The EE, HE, and AF showed the strongest antistaphylococcal activity, whose MIC values ranged from 64 to 256 *μ*g/mL. When these extracts were compared, no statistical difference was observed.

According to Sartoratto et al. [29] a strong activity is for MIC values between 50 and 500 **μ**g/mL, moderate activity for MIC values between 600 and 1500 **μ**g/mL, and weak activity for MIC values above 1500 **μ**g/mL [29]. In comparison to literature data, the HE, AE, EE, EAF, and AF, whose MIC ranged from 64 to 512 **μ**g/mL, have a strong antimicrobial activity against the *Staphylococcus aureus* strains tested.

High phenol contents were found in the EE, HE, and AF. These compounds can be directly related to antimicrobial activity. These results corroborate with those described by Cowan, 1999 [30], and Okoro et al. [31], who attribute a positive correlation between tannin or flavonoid contents and antimicrobial activity.

Tannins are compounds that, when in the aqueous system, form colloidal solutions and their antimicrobial activity is due to binding to proteins and adhesins, inhibiting enzymes, the complexation with the cell wall and metal ions, and disruption of the plasmatic membrane. However, the flavonoids have the ability to complex with protein and bacterial cells forming irreversible complexes mainly with nucleophilic amino acids [30].

**Figure 2:**
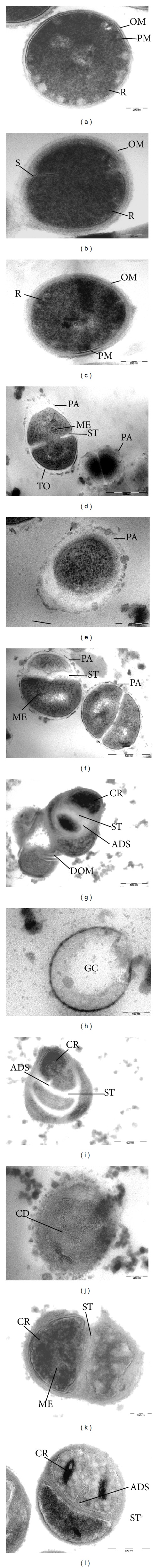
Electron transmission microscope images taken of *S. aureus* ATCC-33591 cells not treated; (a)–(c) show distinct OM: outer membrane, PM: plasma membrane, R: ribonucleus, and S: septa of the bacteria. Treated with hydroalcoholic extract of *Hymenaea stigonocarpa* in half of minimum inhibitory concentration; (d)–(f) show indentation and hole formation appearances (PA), mesossomes (M), thickening of the outer membrane (TO), and septum thickness distribution (ST). Treated with minimum inhibitory concentration; (g)–(k) show condensing of the ribonucleus (CR), detachment of outer membrane (DOM), thickness distribution septa (ST) and aberrant division septa (ADS), ghost cells (GC), mesossomes (ME), and coagulated DNA (CD). Magnification of (a), (b), (c), (e), (j), and (k) is 30000x and Magnification of (d), (f), (g), (h) and (l) are 65000x.

The action of the phenolic compounds present in the hydroalcoholic extract can be visualized by electron transmission microscopy (TEM) on *S. aureus* ATCC 33591, as in Figure 2.

### 3.4. Ultrastructural Alterations

The cytological alterations caused by hydroalcoholic extract at 1/2 MIC included partially digested cell-wall fragments exfoliated from the bacterial surface, exposition of the patches in the cytoplasmic membrane (Figures 2(d)-2(e)), the absence of the cell wall or detachment from the cytoplasmic membrane (Figure 2(e)), the thickening of the outer membrane TO (Figure 2(e)), and abnormal septa (Figures 2(d), 2(f), 2(i), 2(k), and 2(l)), which appears to have been caused by some dysfunction during the cell division.

The appearance of a thickening of the outer membrane (TO) and a condensation of the ribonucleus (CR) into bacteria indicate defensive mechanisms to maintain the osmotic pressure after hydroalcoholic extract action. Bacteria also produce more peptidoglycan to protect themselves and develop mesossomes (ME), implicated in the cellular respiration [32].

The failure to insert the peptidoglycan caused in the outer membrane the hole formation appearances (PA) (Figures 2(d), 2(e), and 2(f)). This alteration was caused to the difference in osmotic pressure between the outer and inner surroundings of the bacteria. If this event was to be prolonged, it would have caused bacterial lysis [33].

The minimal inhibitory concentration (256 *μ*g/mL) of the hydroalcoholic extract was able to provoke alterations in several cell structures. Figures 2(g)–2(l) showed cell membranes and cell walls being disrupted and damaged, resulting in a release of cell materials into the cytoplasm (Figures 2(g) and 2(h)). Alterations in the intracellular components such as nucleic acids, proteins, aggregated and branched ribosomes as well as the presence of multiple septa were visualized in Figures from 2(i) to 2(l). The term of DNA coagulated effects (DC) means that the function of DNA gyrase was stopped from fulfilling its main core in supercoiling the DNA during replication.

Tannins main compounds present in hydroalcoholic extract are able to bind to the membrane to create connections between the double layers and initiate their interaction and adhesion. This process is accompanied by the decrease of the membrane potential and interlayer spacing.

In this study, the cytological changes observed by TEM at different concentrations of the hydroalcoholic extract from *Hymenaea stigonocarpa* stem barks are supporting evidence of its antibacterial action against *Staphylococcus aureus*.

## 4. Conclusion

The results obtained in this study indicate that the *Hymenaea stigonocarpa* stem barks have flavonoids and tannins and these compounds are possibly the reason for the antimicrobial activity against Gram-positive *cocci*.

*Hymenaea stigonocarpa *might be an interesting alternative therapy for infectious diseases caused by multidrug resistant microorganisms. In addition, more studies including toxicity tested *in vivo* need to be conducted on this plant before therapeutic treatments are implemented.

## Figures and Tables

**Table 1:** Yields, total phenols, and tannins from *Hymenaea stigonocarpa* extracts and fractions.

Extracts/fractions	Yield (%)	Total phenol content	Total tannin
Cyclohexane (CE)	0.35 ± 0.02	0.00	0.00
Ethyl acetate (EAE)	2.65 ± 0.22	6.63 ± 0.13	1.82 ± 0.17
Ethanolic (EE)	11.99 ± 0.75	7.26 ± 0.04	2.76 ± 0.10*
Aqueous (AE)	2.09 ± 0.06	4.95 ± 0.02	1.46 ± 0.06
Hydroalcoholic (HE)	15.49 ± 056	6.96 ± 0.09	2.96 ± 0.08*
Aqueous (AF)	57.00 ± 5.00	6.16 ± 0.03	2.71 ± 0.05*
Ethyl acetate (EAF)	22.10 ± 2.20	7.12 ± 0.03	2.00 ± 0.04

Yield expressed in percentage (%).

Total phenols and total tannin contents expressed in mg GAE/g of dried samples.

*Means are not significantly different (*P* < 0.05).

**Table 2:** Minimal inhibitory concentrations of the extracts and fractions from *Hymenaea stigonocarpa* against Gram-positive*  cocci*.

Gram-positive* cocci *	Origin	Minimal inhibitory concentrations (*μ*g/mL)									Resistance phenotype
		CE	EAE	EE	AE	HE	AF	EAF	OXA	TEI	
*Staphylococcus aureus *ATCC 25293	ATCC	512	512	256	512	128	128	128	0.12	—	Control strain
*Staphylococcus aureus *ATCC 33591	ATCC	1024	512	256	512	256	256	512	64	—	Control strain
*Staphylococcus aureus *LFBM 05	Hemoculture	1024	512	256	512	256	125	512	32	—	AMP, AZI, CFO, PEN
*Staphylococcus aureus *LFBM 11	ND	512	512	128	128	128	128	256	1.0	—	PEN
*Staphylococcus aureus *LFBM 13	Vaginal secretion	512	512	512	512	128	64	512	1.0	—	PEN
*Staphylococcus aureus *LFBM 15	Sputum	512	512	128	512	64	64	256	0.12	—	PEN, GET, ERI, CLI
*Staphylococcus aureus *LFBM 16	Hemoculture	512	512	256	512	256	125	256	0.12	—	AMP, AZI, CFO, PEN
*Staphylococcus aureus *LFBM 25	Hemoculture	512	512	256	512	256	256	256	256	—	PEN, GET, ERI, RIF, CLI
*Staphylococcus aureus *LFBM 26	Hemoculture	512	512	256	512	64	64	512	256	—	AMC, AMP, CFO, CIP
*Staphylococcus aureus *LFBM 28	Tracheal secretion	1024	512	256	512	256	128	512	16	—	AMC, CIP, CFO, GET
*Staphylococcus aureus *LFBM 29	Hemoculture	1024	512	256	512	256	256	512	8.0	—	AMC, AMP, CFO, PEN
*Staphylococcus aureus *LFBM 30	Tracheal secretion	1024	512	256	512	256	256	512	16	—	AMC, AMP, CFO, CIP
*Enterococcus faecalis *LFBM 02	Hemoculture	1024	512	256	256	256	256	512	—	8.0	CIP, PEN, ERI, IMI, TET
*Enterococcus faecalis *LFBM 07	Hemoculture	1024	512	256	512	512	256	512	—	8.0	CIP, PEN, ERI, IMI, TET
*Enterococcus faecalis *LFBM 08	Urine	1024	512	256	512	256	256	512	—	0.25	None antibiotic
*Enterococcus faecalis *LFBM 18	Hemoculture	1024	512	256	512	256	256	512	—	4.0	CIP, PEN, ERI, IMI, TET

ATCC: American Type Culture Collection; CE: cyclohexanic extract; EAE: ethyl acetate extract; EE: ethanolic extract; AE: aqueous extract; HE: hydroalcoholic extract; AF: aqueous fraction; EAF: ethyl acetate fraction; OXA: oxacillin; AMP: ampicillin; AZI: azithromycin; CFO: cefoxitin; CIP: ciprofloxacin; GET: gentamicin; PEN: penicillin; CLI: clindamycin; ERI: erythromycin; IMI: imipenem; RIF: rifampin; TET: tetracycline; LFBM: Laboratório de Fisiologia e Bioquímica de Microorganismos; ND: not determined.
